# Endoglin inhibition leads to intussusceptive angiogenesis via activation of factors related to COUP-TFII signaling pathway

**DOI:** 10.1371/journal.pone.0182813

**Published:** 2017-08-31

**Authors:** Ruslan Hlushchuk, Beata Styp-Rekowska, Jehona Dzambazi, Monika Wnuk, Uyen Huynh-Do, Andrew Makanya, Valentin Djonov

**Affiliations:** 1 Institute of Anatomy, University of Bern, Bern, Switzerland; 2 Department of Nephrology and Hypertension, Inselspital Bern, Bern, Switzerland; 3 Department of Veterinary Anatomy and Physiology, University of Nairobi, Nairobi, Kenya; Universita degli Studi di Bari Aldo Moro, ITALY

## Abstract

Angiogenesis is a highly coordinated, extremely complex process orchestrated by multiple signaling molecules and blood flow conditions. While sprouting mode of angiogenesis is very well investigated, the molecular mechanisms underlying intussusception, the second mode of angiogenesis, remain largely unclear. In the current study two molecules involved in vascular growth and differentiation, namely endoglin (ENG/CD105) and chicken ovalbumin upstream promoter transcription factor II (COUP-TFII) were examined to unravel their specific roles in angiogenesis. Down- respectively up-regulation of both molecules tightly correlates with intussusceptive microvascular growth. Upon ENG inhibition in chicken embryo model, formation of irregular capillary meshwork accompanied by increased expression of COUP-TFII could be observed. This dynamic expression pattern of ENG and COUP-TFII during vascular development and remodeling correlated with formation of pillars and progression of intussusceptive angiogenesis. Similar findings could be observed in mammalian model of acute rat Thy1.1 glomerulonephritis, which was induced by intravenous injection of anti-Thy1 antibody and has shown upregulation of COUP-TFII in initial phase of intussusception, while ENG expression was not disturbed compared to the controls but decreased over the time of pillar formation. In this study, we have shown that ENG inhibition and at the same time up-regulation of COUP-TFII expression promotes intussusceptive angiogenesis.

## Introduction

Intussusception is a mechanism of vascular growth that is supplemental to the process of sprouting angiogenesis. This process allows rapid expansion of incipient capillary plexuses and plays an important role in vascular remodeling under normal and pathological conditions. However, the basic molecular principles are still not yet examined adequately [1–3]. Our previous data indicate that VEGF has a supportive role during initiation of intussusceptive angiogenesis (IA). On the other hand studies with inhibition of VEGF-signaling proved that intussusceptive vascular growth, expansion and remodeling occur mainly in a VEGF-independent manner [4–6]. Additionally, our recent studies indicate that within perfused vascular beds Notch is potential key player inducing IA [7, 8]. Inhibition of Notch signaling resulted in induction of IA with an increased capillary density of more than 50%. Concomitant with the robust IA there was detachment of pericytes from basement membranes, increased vessel leakage and recruitment of mononuclear cells to the sites of pillar-formation. The events were dramatically enhanced when we combined Notch inhibition with injection of bone marrow-derived mononuclear cells. Moreover, numerous studies have demonstrated the involvement of the transforming growth factor beta (TGFβ), its receptors, for example Endoglin, (ENG) and members of the Notch receptor family play a key role in pathogenesis of glomerular diseases [9–12].

ENG is a proliferation-associated cell membrane antigen and is a part of TGFβ receptor complex required for angiogenesis [13–15]. ENG null embryos exhibit a loss of arteriovenous identity and defective vascular smooth muscle cell (vSMC) recruitment [14, 15]. Haploinsufficiency of ENG results in Hereditary Hemorrhagic Telangiectasia (HHT), characterized by a loss of arteriovenous identity and aberrant vSMC incorporation in fragile vessels [14–16]. Venkatesh et al. proposed Notch as a regulator of ENG/TGFβ signaling in endothelium, and Notch suppression of this pathway contributing to loss of paracrine signaling to the SMCs [17]. ENG seems not to be directly involved in signaling, but can modulate TGFβ signaling through the ACVRL1 or ACVRL5 (ALK1/ALK5, activin receptor-like kinase 1 / 5) to promote cell proliferation and migration for instance. ENG can specifically enhance TGFβ1-induced phosphorylation of SMAD 1/5/8 (Small/mothers against decapentaplegic), increase a SMAD 1/5/8 responsive promoter, whereas phosphorylation of SMAD`s supports their translocation to the nucleus to regulate expression of downstream genes [13, 15, 18, 19]. Yangxin Fu et al. have shown that Notch and TGFβ signaling synergistically induce Snail (zinc finger SNAi-a transcriptional repressor) expression in endothelial cells, which is required for EndMT in cardiac cushion morphogenesis [20]. In addition they reported that Notch activation modulates TGFβ signaling pathways in a receptor-activated SMAD (R- SMAD)-specific manner, triggering SMAD1, SMAD2 and SMAD3 [21]. At this level, it seems that interaction or competitive binding with chicken ovalbumin transcription factors occurs.

Chicken ovalbumin upstream transcription factor II is a member of the COUP-TF orphan subfamily of the nuclear receptor superfamily of transcription factors. It has roles in angiogenesis, vascular remodeling and heart development [22, 23]. COUP-TFII is expressed in tissues in all major physiological systems with particularly high levels in the adrenal gland, kidney, ovary, uterus and vas deferens. During arterio-venous differentiation COUP-TFII suppresses neuropilin1 expression, thereby suppressing reception of the VEGF-A signal and activation of Notch signaling [22]. In addition, activation of PI3K/Akt signaling antagonizes promotion of arterial cell differentiation by blocking ERK (Extracellular-signal Regulated Kinase) activation. Thus, COUP-TFII has a critical role in repressing Notch signaling to maintain vein identity [24, 25]. The chicken ovalbumin upstream promoter-transcription factor II seems to be also a major angiogenesis regulator within the tumor microenvironment [26]. Removal of COUP-TFII in adults severely compromised neo-angiogenesis and suppressed tumor growth in xenograft mouse models. In addition, tumor growth and tumor metastasis were also impaired in a spontaneous mammary-gland tumor model in the absence of COUP-TFII. It was shown that COUP-TFII directly regulates the transcription of Angiopoietin-1 in pericytes to enhance neoangiogenesis. Importantly, provision of Angiopoietin-1 partially restores the angiogenic defects exhibited by the COUP-TFII–deficient mice, which supports the notion that COUP-TFII controls Angiopoietin-1/Tie2 signaling to regulate tumor angiogenesis [26, 27].

There are several findings supporting the hypothesis of tight interplay between TGFβ/ENG signaling pathway and COUP-TFII. Indication for link between COUP-TFII and ENG was described by Mancini et al. in theirs study [16]. Atypical arterial expression of the venous specific COUP-TFII was found in ENG null embryos [16]. ENG re-expression in endothelial cells restored normal COUP-TFII expression. Furthermore, ablation of COUP-TFII in endothelial cells enables veins to acquire arterial characteristics, including the expression of arterial markers NP-1 (neuropillin-1) and Notch signaling molecules, and the generation of haematopoietic cell clusters. COUP-TFII mutants are defective in remodeling the primitive capillary plexus into large and small microcapillaries. Moreover, ectopic expression of COUP-TFII in endothelial cells results in fusion of veins and arteries in transgenic mouse embryos in a manner similar to that found in HHT where there is inappropriate fusion of arterioles with venules [16]. Korten et al. suggested a repression of Notch pathway by COUP-TFII as well. In the latter study, endothelial-specific overexpression of COUP-TFII in transgenic mouse embryos exhibited large, fused, disorganized vessels without arteriovenous distinction and reduced expression of genes of the Notch pathway [28].

The study of Qin et al. [27] concerning whether COUP-TFII could interact directly with any of SMAD proteins from ENG signaling pathway showed indeed that COUP-TFII was strongly associated with SMAD4 in cells and in tumour specimens. Furthermore, the data indicated that COUP-TFII sequestered SMAD4 from binding to TGFβ-target gene promoters in cells and in tumours containing higher levels of COUP-TFII [27, 29].

There are several findings supporting the hypothesis of tight interplay between TGFβ/ENG signaling pathway and COUP TFII. In this case ENG may play a role in stimulation of intended kind of angiogenesis in common with/ or via COUPTFII and in this way also contribute to development of cancer and other angiogenesis-associated diseases which IA is culprit. Here we provide evidence that endoglin inhibition influences expression of the chicken ovalbumin upstream promoter transcription factor II and is in this connection attended by intussusceptive angiogenesis.

## Material and methods

### Angiogenic assays in vivo

#### Chicken model

Fertilized White Leghorn eggs obtained from Wüthrich Brüterei AG (Belp, Switzerland) were incubated at 37°C and 60–90% relative humidity for 72 h, corresponding to stage HH10–HH12 of embryonic development. Subsequently, eggs were opened and embryos placed into the petri dishes. After approx. 1 h targeted treatments were performed by applying the required agents directly on the selected quadrat of the area vasculosa. All treatments were applied in a final volume of 40 μl. Assays involving antibodies and siRNA were carried out with amounts selected as optimal according to our previous studies [8]. After addition of stimuli, embryos were incubated for an additional period of 6, 12, 24 and 48h. For imaging Lectin-conjugated FITC was injected into the veins and observed under stereo microscope. Then, samples were excised and fixed in 2% paraformaldehyde buffered in 0.1 M phosphate buffer, pH 7.1 or collected in liquid nitrogen. A total of at least 5 experiments were carried out and on average, 10 embryos were tested for each treatment, discarding non-viable embryos.

#### Rat model (Wnuk et al. [12])

All procedures were conducted according to the National Institutes of Health guidelines for the care and use of laboratory animals and with the approval of the local animal ethics committee (Bernese Cantonal Veterinary Office). Male Wistar rats weighing 170 to 190 g at the beginning of the study were obtained from Charles River Laboratories, Sulzfeld, Germany. Acute Thy1.1 glomerulonephritis was induced by a single intravenous injection of monoclonal OX7 antibody at the dose of 1 mg/kg of body weight. Rats were sacrificed on days 2, 5, 9, 14, and 21. Animals receiving the same volume of saline served as control.

### Reagents

Human-specific CD105 (BD), Chicken specific antibody (kind donation from Prof. Bernabeu, Madrid) and siRNA (Santa Cruz (h): sc-35302) were used according to the manufacturer’s protocols and adjusted to experimental setup. Prof. Tsai (Baylor College of Medicine, Houston) kindly donated the COUP-TFII plasmid used in transfection experiments.

### RNA isolation and real-time reverse transcription-PCR

Total RNA was extracted from the treated lower right quadrant of the area vasculosa, treated with the respective inhibitors and also from non-inhibited samples, by using RNeasy Mini kit (Qiagen, Basel, Switzerland). The RNA concentrations were determined spectrophotometrically. cDNA was synthesized by reverse transcription (1 hour at 37°C) using 2 μg of total RNA, 1 μg of the oligo-dT primers and 4U of ImPromp-II reverse transcriptase (Promega, Wallisellen, Switzerland). cDNA concentrations were determined using the PicoGreen dsDNA-quantification reagent (Invitrogen, Basel, Switzerland). The following primer/probe kits were purchased QuantiTest Primer Assay from Qiagen: ENG (QT01489397/), Notch2 (QT01504986/), COUPTFII (NR2F2; QT00058492) VEGFA (QT00593327), VEGFR2 (KDR). Quantitative PCR was performed using Real Time PCR (Applied Biosystems). The fold change in expression levels of cDNA were determined from the ΔCt values obtained as compared to ΔCt values of control samples GAPDH (QT00588973/) was used as housekeeping gene.

### Immunofluorescence staining

Area vasculosa samples were obtained from White Leghorn chicken embryos (Wütherich GmbH). Sections were fixed by immersion in 2% buffered formalin for immunostaining. After dehydration, pieces were embedded in paraffin and 3–5 μm sections were cut and mounted on glass slides and stained with the targeted antibodies as well as Hoechst dye (Sigma) according to the manufacturer’s protocols. The antibodies used: anti-COUP-TFII (Thermo Fisher Scientific Inc., Cat. No PA1-23589), anti-ENG (kind donation from Prof. Bernabeu, Madrid). Labelling was revealed by fluorescence microscopy using a stereo (Leica FX) and confocal microscope (Leica 510Z).

### RT^2^ Profiler^™^ PCR Array

RT^2^ Profiler PCR Arrays (SABioscences/Qiagen AG, Switzerland) are designed to analyze a panel of genes related to a disease state or biological pathway. RT^2^ Profiler PCR Arrays used, were provided as 96-well plates, contained primer assays for 84 TGFβ signaling pathway or transcription factor related genes and 5 housekeeping genes. In addition, one well contained a genomic DNA control, 3 wells contained reverse-transcription controls, and 3 wells contained positive PCR controls. Assays for 5 housekeeping genes included in the arrays enable normalization of data. The genomic DNA control (GDC) is an assay that specifically detects nontranscribed genomic DNA contamination with a high level of sensitivity. The reverse-transcription control (RTC) is an assay that tests the efficiency of the reverse-transcription reaction performed with the RT2 First Strand Kit by detecting template synthesized from the kit’s built-in external RNA control. The positive PCR control (PPC) consists of a predispensed artificial DNA sequence and the assay that detects it. This control tests the efficiency of the polymerase chain reaction itself. The normalization was carried out via geometric averaging for all data.

### Morphometry

Evaluation of vascular parameters was accomplished with TEM Imaging Platform software (iTEM). Micrographs were obtained from normally developing area vasculosa in order to obtain baseline data and a normal growth curve and from treated samples. From the each experimental group, micrographs were taken from inhibited samples at time points 6 h, 12 h, 24h and 48 h. The vascular tree of the lower right quadrant was traced in a user-driven way. We took pictures with the Leica M205FA microscope (magnification 160.4x/0.1615μm pro pixel). The same procedure was repeated with ENG siRNA (diluted after SantaCruz protocol) for 24 and 48 hours. By dividing the pictures in 225 x 200 μm areas, we chose three for area of interest. For Vessel Area Density an overlay with a 20 x 20 μm measuring grid was used. Every cross point that was on a vessel was detected. Finally the sum of the detected cross points in the area of the interest was divided by the sum of the containing cross points (30).

### Vascular casting

For the injection of blood vessels polyurethane elastomer PU4ii dilutions according to the manual was used. Shortly before injection, the hardener-L (ratio 100:18 (v/v), vasQtec, Switzerland, processing time 25 min) was added. The resulting mixture was thoroughly stirred, avoiding formation of air inclusions. The resulting low viscosity elastomer was transferred into a syringe and injected into the vessels. The dissection of vessels started after 5 days at room temperature. After treatment with KOH, corrosion casts were mounted on aluminum stabs and sputtered with 10 nm gold. The specimens and their pattern of vasculature and the condition of the endothelial tissue were then studied with a Phillips XL-30 FEG scanning electron microscope.

### Transmission electron microscopy

Treated samples obtained from the area vasculosa and controls harvested at different time points were fixed in 2.5% glutaraldehyde solution buffered with 0.03 M potassium phosphate (pH 7.4, 370 mOsm). They were then postfixed in 1% OsO4 (buffered with 0.1 M sodium cacodylate–pH 7.4, 370 mOsm), dehydrated in ascending concentrations of ethanol, and embedded in epoxy resin. For light microscopy, 1μm-thick sections were prepared using glass knives and stained with Toluidine Blue. For transmission electron microscopy, 80 to 90 nm-thick sections were prepared and mounted on copper grids coated with Formvar (polyvinyl formal; Fluka, Buchs, Switzerland). They were stained with lead citrate and uranyl acetate prior to viewing in a Philips EM-400 electron microscope [30].

### Quantification and statistics

Images were analyzed and quantified using Cell and Image J software. Data were presented as mean ± SD of representative experiments. Statistical significance was calculated using the student’s t test. (*p<0.05; **p<0.01; ***p<0.001).

## Results

### Blocking ENG induces intussusceptive angiogenesis and inhibits maturation of the vascular network in area vasculosa

**Fig 1:**
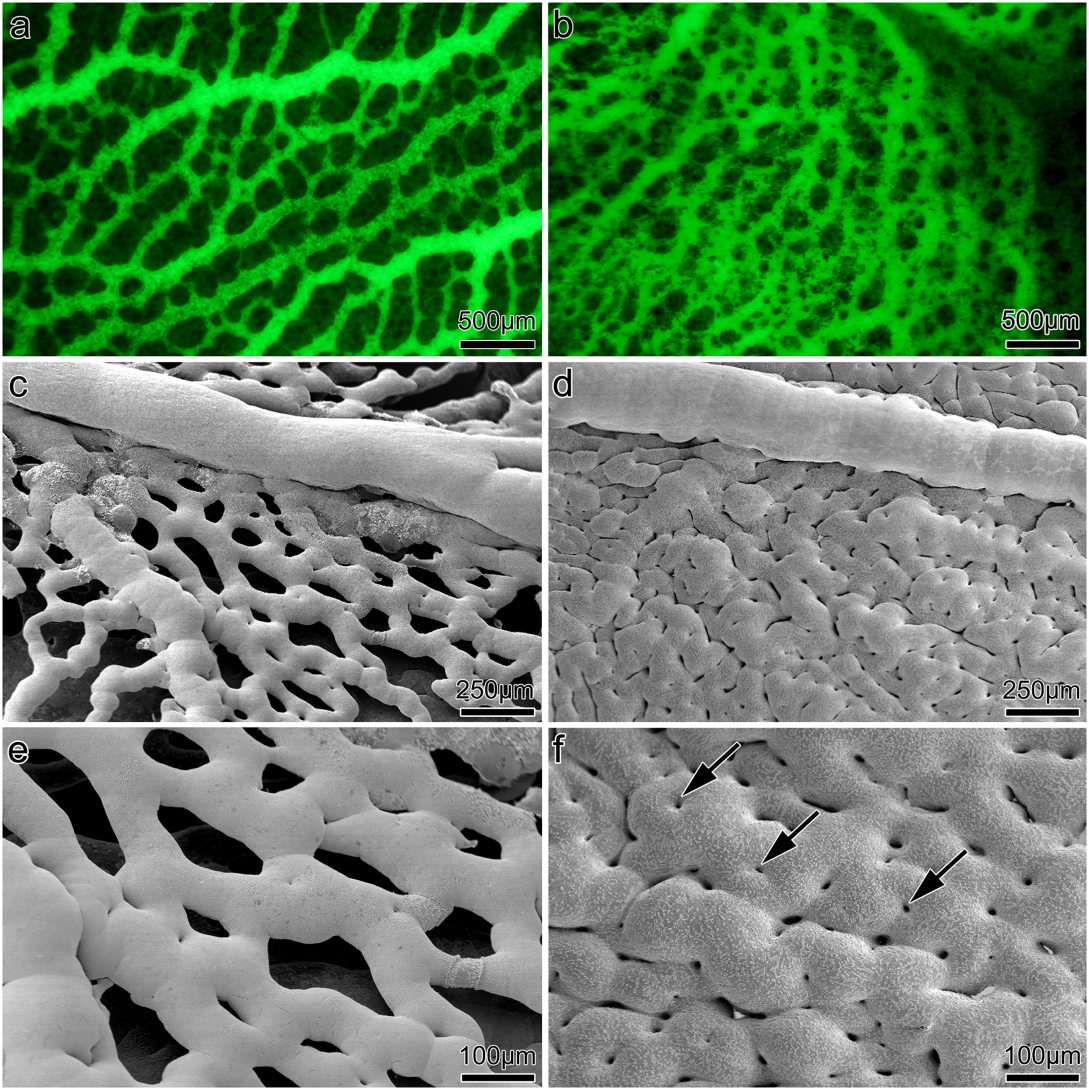
Area vasculosa of developing chicken embryos. Left panel control, right panel ENG inhibition by endoglin antibody. a,b: Lectin FITC conjugated staining indicated very dense capillary plexus 24h after treatment with antibody. c-f: Vascular casts revealed an extreme example of very crowded, sponge-like capillary plexus after ENG inhibition. The multiple tissue pillars appear as a “holes” within the plexus (arrows). e’ and f’ parts of c and d respectively.

We investigated the effects of ENG inhibition on blood vessel development and maturation in the area vasculosa of chicken embryos. The visualization was carried out by injection of lectin-FITC conjugated solution into a vein 6 h, 12 h, 24 h and 48 h after treatment (Fig 1a and 1b). The pattern of vasculature has been additionally studied by scanning electron microscopy (SEM) of PU vascular casts (Fig 1c–1f). The SEM data provide in addition to FITC staining better resolution and closer view within the 3D vascular morphology. Sponge-like structures appeared in capillary plexuses 24 h after treatment with ENG specific antibody (CD105 antibody, 2μg/ml) and ENG siRNA. Anti-ENG treated embryos developed a dense sheet of vessels, with a significant increase in the vascularized area compared to the timed controls (Fig 1b and 1d). In contrast to the controls, the number of pillars, hallmarks of intussusceptive angiogenesis increased remarkably in treated embryos. We also observed relatively often intussusceptive pillars on the arterial side, whereas during normal conditions it is predominant on the venous side of vasculature. The development of the vascular network of the controls did not show any abnormalities Fig 1a and 1c).

**Fig 2:**
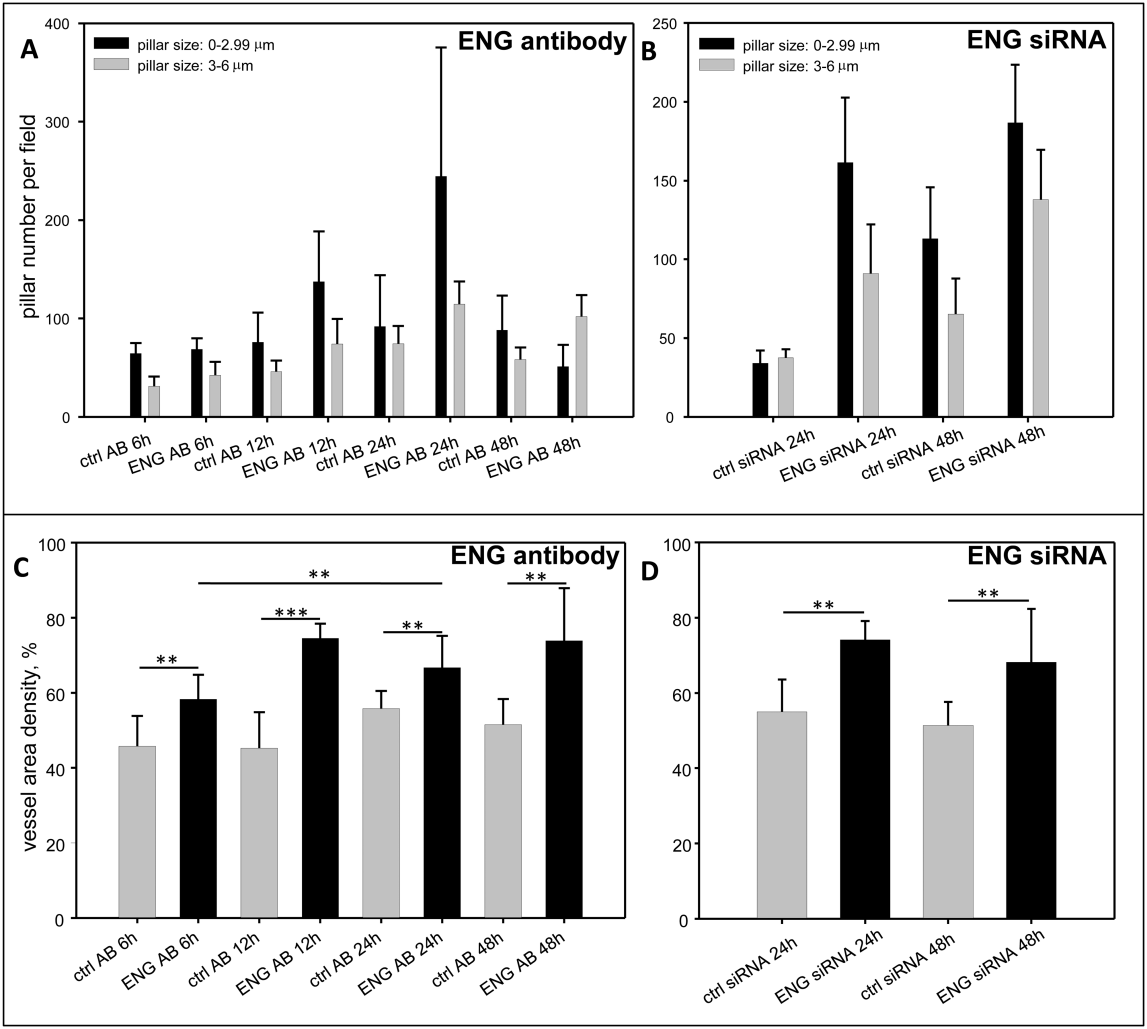
Quantification of pillar number (a-b) and relative vascular density (c-d) in embryos treated with an anti-ENG antibody (a and c) and with ENG siRNA (b and d) compared to the controls. Significant increase in the number of small pillars (diameter ≤ 2.99μm) was observed in the samples treated with an anti-ENG antibody or with ENG siRNA. In the treated groups vessel area density followed the same correlation indicating a higher number of vessels in the observed field. In controls the number of bigger holes (>3μm) was much higher than in the treated group, which indicated advanced stage of vascular maturation A total of at least 5 experiments were carried out and on average 10 embryos were tested for each treatment.

Measurements of the pillar number, vascular area and vascular densities showed a significant increase after treatment with the antibody (Fig 2a and 2c). These results were confirmed with measurements of vascular changes after ENG siRNA treatment (Fig 2b and 2d). The effects of ENG inhibition by specific antibody were reaffirmed by real time PCR as shownfurther below.

**Fig 3:**
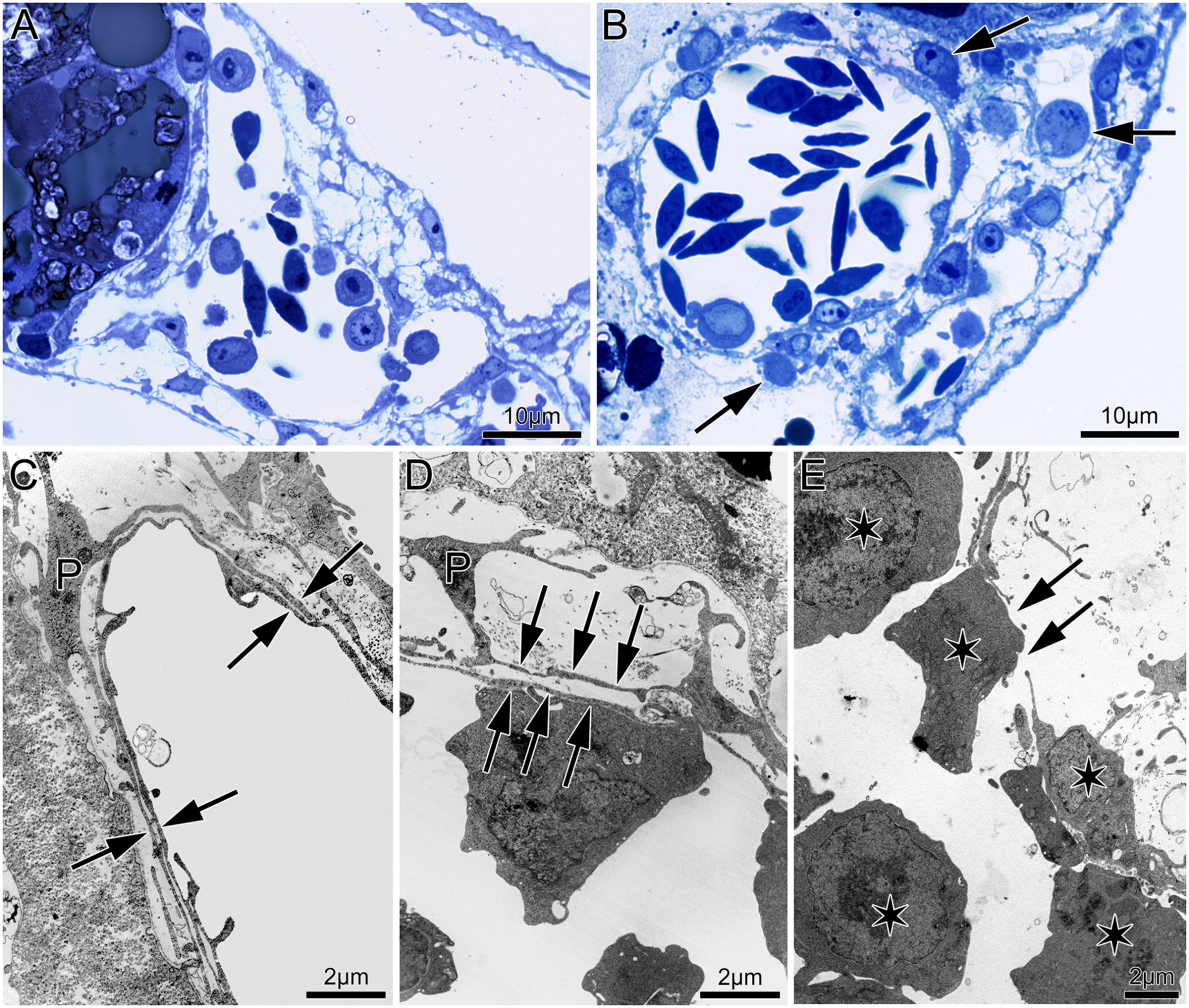
Light and TEM microscopy of area vasculosa controls (a and c) vs 24h inhibition by ENG antibody (b d and e). Treated samples showed extravasation of mononuclear cells (arrows) TEM revealed pericyte (P) detachment with significantly increased endothelial–pericyte layer (double arrows; d). For comparison please see c. Often, disrupted endothelium (arrows in e) serves as an thelium (arrows in othelial mononuclear cells (arrod by asterisks.

Morphological investigations of the treated samples revealed intraluminal accumulation of mononuclear cells which attach to the vascular wall and in a subsequent step extravasate. The latter event indicates most likely chemoattractant capability and/or increased vascular permeability (Fig 3a and 3b). Zoom in demonstrated clearly pericyte detachment with significantly increased periendothelial space; the endothelium appears interrupted with smaller and larger gaps with extravasating mononuclear cells (Fig 3c and 3d)

### TGFβsignaling pathway is disturbed after ENG blockage

**Fig 4:**
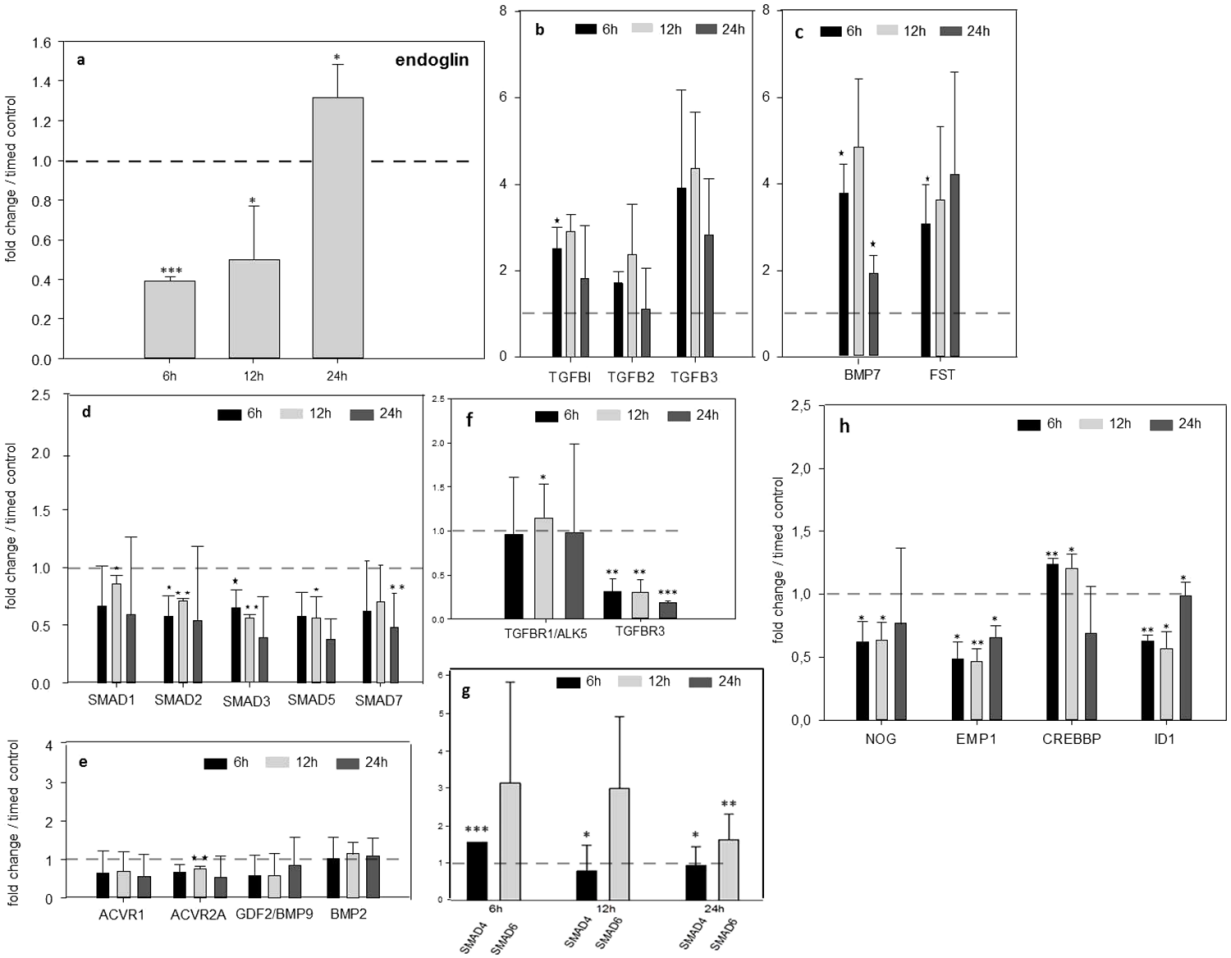
Real-time RT-PCR assessment of ENG expression and gene expression alterations of TGFβ and ENG downstream signaling molecules and transcription factors. **a**: After treatment with anti ENG specific antibody the ENG gene expression was significantly down-regulated after 6 h, 12 h in comparison to the controls without treatment (dashline). After 24 h gene expression was almost restored to basic level. Repeated application of ENG specific antibody led to dramatically decreased expression around 48h after. **b** and **c**: Upregulated expression of three primary of types TGFβ as well BMP7 and follistatin (FST). **d and g**: Gene expression profile of SMADs proteins 1–7 in time-course after ENG inhibition. **e**:, BMP-related molecules ACVR1 and ACVR2A as well as bone morphogenic proteins 2 and 9-epression profiles demonstrated reduced expression after ENG inhibition. **f**: TGFβR1 (ALK5) and TGFβR3 factors interacting with ENG were down-regulated. **h**: ENG downstream signaling molecules NOG, EMP1, CREBBP and ID1 showed significant decreased expression. Usually Notch cooperating SMAD3 expression was down-regulated (**d**) also in comparison to SMAD6 (**g**). All: pooled sampling (n = 5), analyzed in triplicate.

Beside examination of morphological changes after treatment with anti-endoglin antibody in area vasculosa of chicken embryos, real-time-PCR was carried out to analyze endoglin gene expression profile over the time (Fig 4a). Here, endoglin was significantly down-regulated after 6h, 12h in comparison to the controls without treatment. After 24h gene expression was almost restored to basic level. Since ENG takes a part in TGFβ signaling, we examined if TGFβ signaling downstream of ENG is involved in alterations in the developing vasculature 6 h, 12 h and 24 h after treatment with ENG specific antibody (Fig 4b–4h). ENG binding partners TGFβ receptor 1 as well TGFβ receptor 3 showed reduced expression after inhibition of ENG (Fig 4f). SMAD7, inhibitor of kinases activity and known as opponent to COUPTF II [31], was down-regulated too (Fig 4d) Expression of ACVR1, signal transducers to BMPs ACVR2A, which binds to BMPs and recruits R-SMADs was decreased as well (Fig 4e) Almost all molecules involved in TGFβ signaling downstream as NOG, EMP1, CREBBP, ID1 and at the level of transcription such as SMAD1, SMAD2, SMAD3, SMAD5 had reduced expression 24 h after treatment of the area vasculosa with ENG antibody in comparison to controls (Fig 4d and 4h). Low amounts of SMAD4 and concurrent high levels of SMAD6 pointed to strict interactive regulation related to ENG inhibition (Fig 4g). Contemporaneous expression of three primary types of TGFβ as well as BMP7 and follistatin (FST), inhibitor of activins, were significantly high (Fig 4b and 4c).

### ENG signaling inhibition leads to increase and alteration of COUP-TFII gene expression

**Fig 5:**
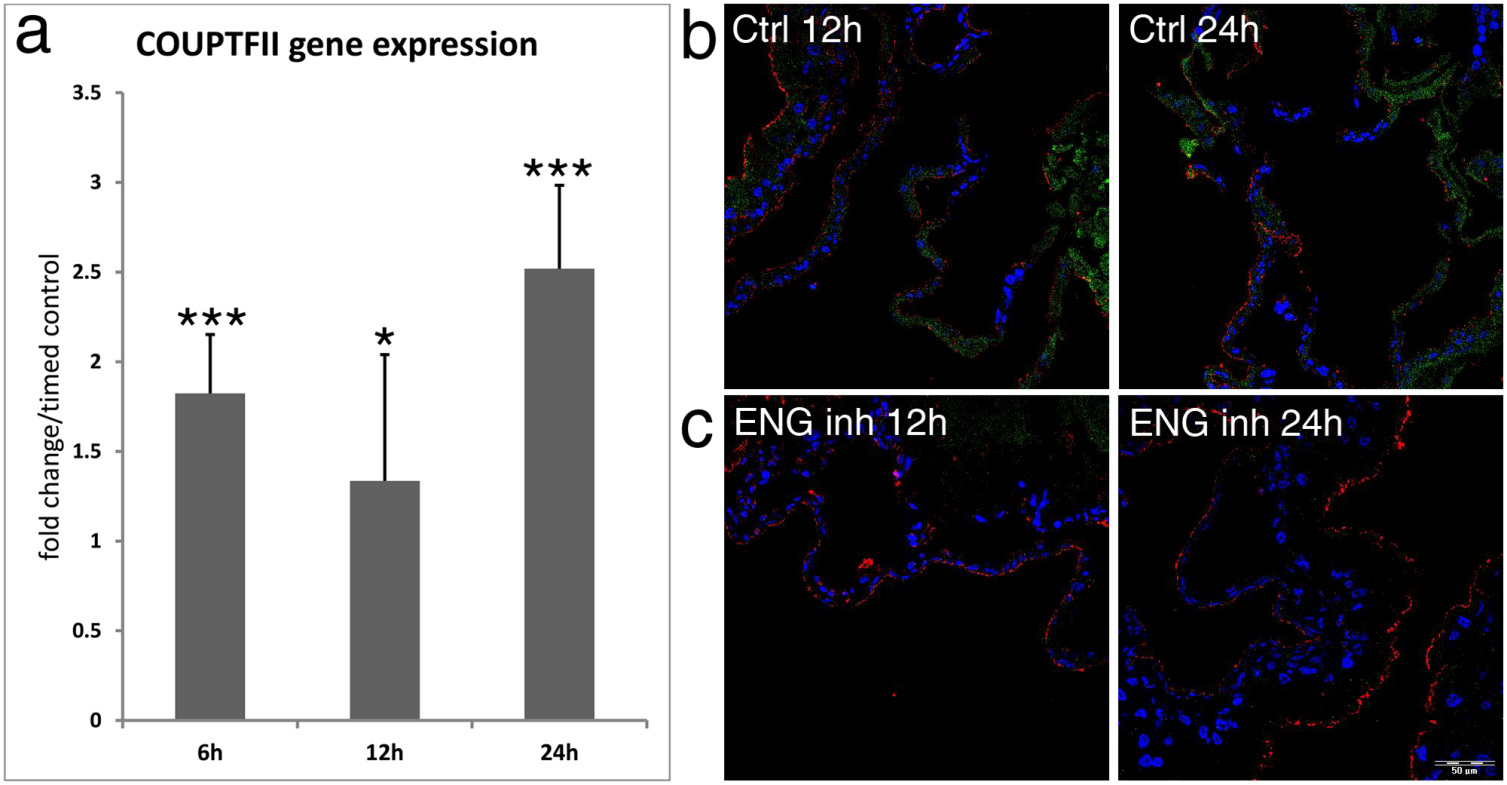
a: Time-course of COUP-TFII gene expression in area vasculosa after inhibition by anti-ENG antibody (pooled sampling (n = 5, analyzed in triplicate). b-c: Immunohistological detection of COUP-TFII expression in the area vasculosa. Samples from control (b) and treated (c) samples shown alterations of COUPTFII localization in the vasculature 24h after blocking endoglin. ENG: green (FITC); COUP-TF-II: red (TRITC); Nuclei: blue (Hoechst).

As next we examined COUP-TFII expression profile after ENG inhibition to determine if ENG and COUP-TFII converge on common pathways. For this purpose we carried out a real time-PCR after 6h, 12h and 24h of endoglin inhibition (Fig 5a). Having demonstrated changed expression of ENG (Fig 4a) and COUP-TFII (Fig 5a) on the RNA level, we analyzed the alteration on protein level by immunofluorescence. As shown in Fig 5b and 5c, ENG and COUP-TFII can be detected in the control animals, but only COUP-TFII appeared after blockage of ENG with specific antibody.

### Overexpression of COUPT-FII leads to rapid morphological changes comparable to ENG inhibition

**Fig 6:**
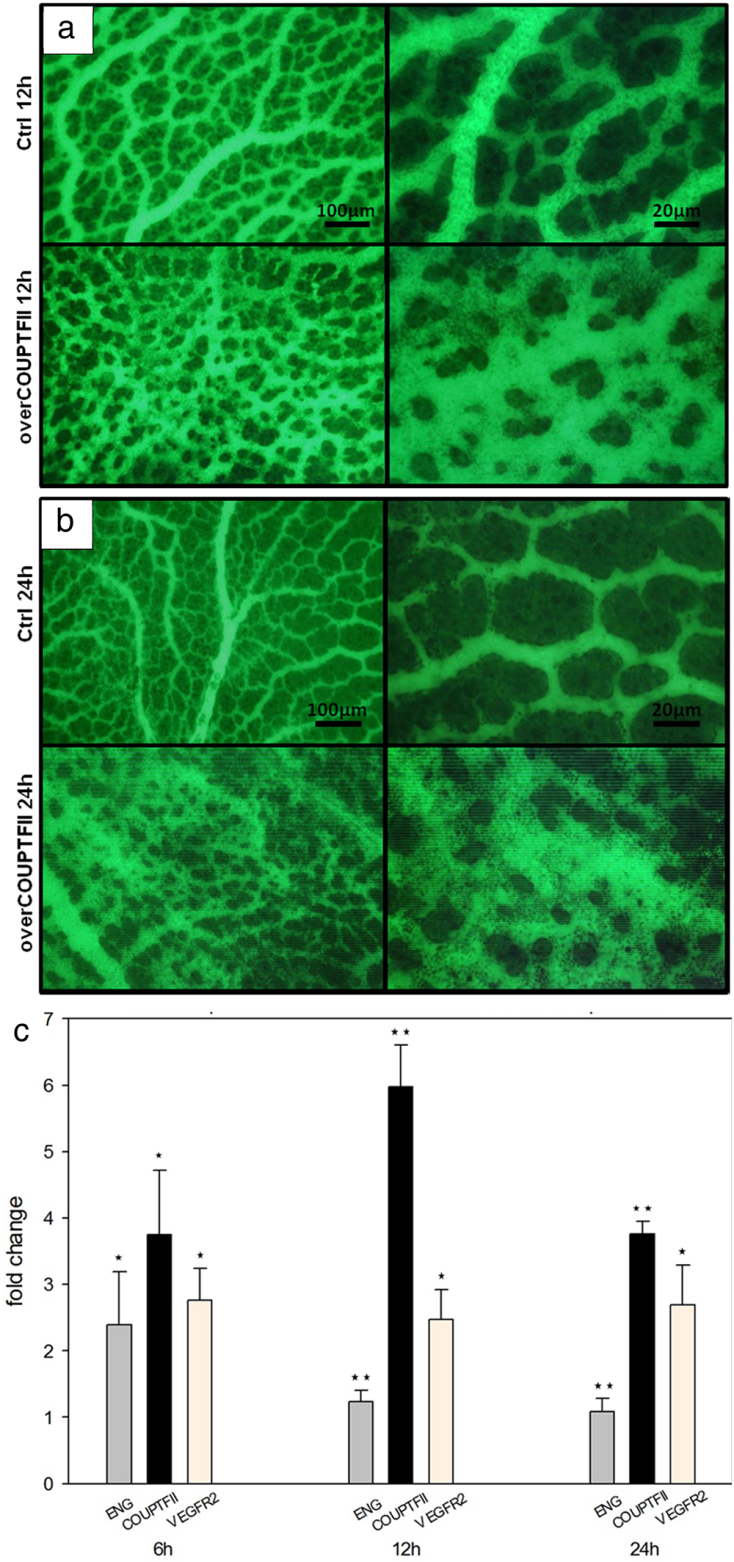
Blood vessel visualization. (a and b, FITC-Lectin conjugated staining) in the area vasculosa 12h and 24h after transfection with COUP-TFII plasmid revealed denser vascular plexus similar to one after ENG inhibition in Fig 1. Gene expression profile (c) after treatment with COUP-TFII plasmid. Time-dependent changes in expression of COUP-TFII in the chicken embryo area vasculosa were associated with down-regulation of ENG (pooled sampling, at least 5 samples analyzed in triplicate).

To examine to what degree COUP-TFII is involved in IA, we transfected 3-day-old chicken embryos with COUP-TFII plasmid. When the transfected embryos were compared with control, we found a perfect correlation between COUP-TF expression and IA (Fig 6a, 6b and 6c) detecting increased number of pillars and significant higher vascular density as compared to the controls.

### COUPT-FII is up-regulated during intussusception phase in rat kidney recovering from acute Thy.1 nephritis

To prove the interplay between ENG and COUP-TFII in mammalian model, we used a model of glomerular injury in rats caused by injection of an anti-Thy1.1 antibody. Recovery of injured glomeruli occurs via intussusceptive angiogenesis and involves molecules connected to the Notch pathway [4]. The upregulation of COUP-TFII (Fig 7.2) started at day 5 after disease induction. From day 9 the expression of COUP-TFII seems to decrease slightly due to natural variability but still was higher than of ENG and Notch2 compared to the healthy controls. The data so far confirm results obtained in the chicken area vasculosa, still further work is needed to elucidate the whole mechanism of COUP-TFII upregulation and action in this pathological situation.

**Fig 7:**
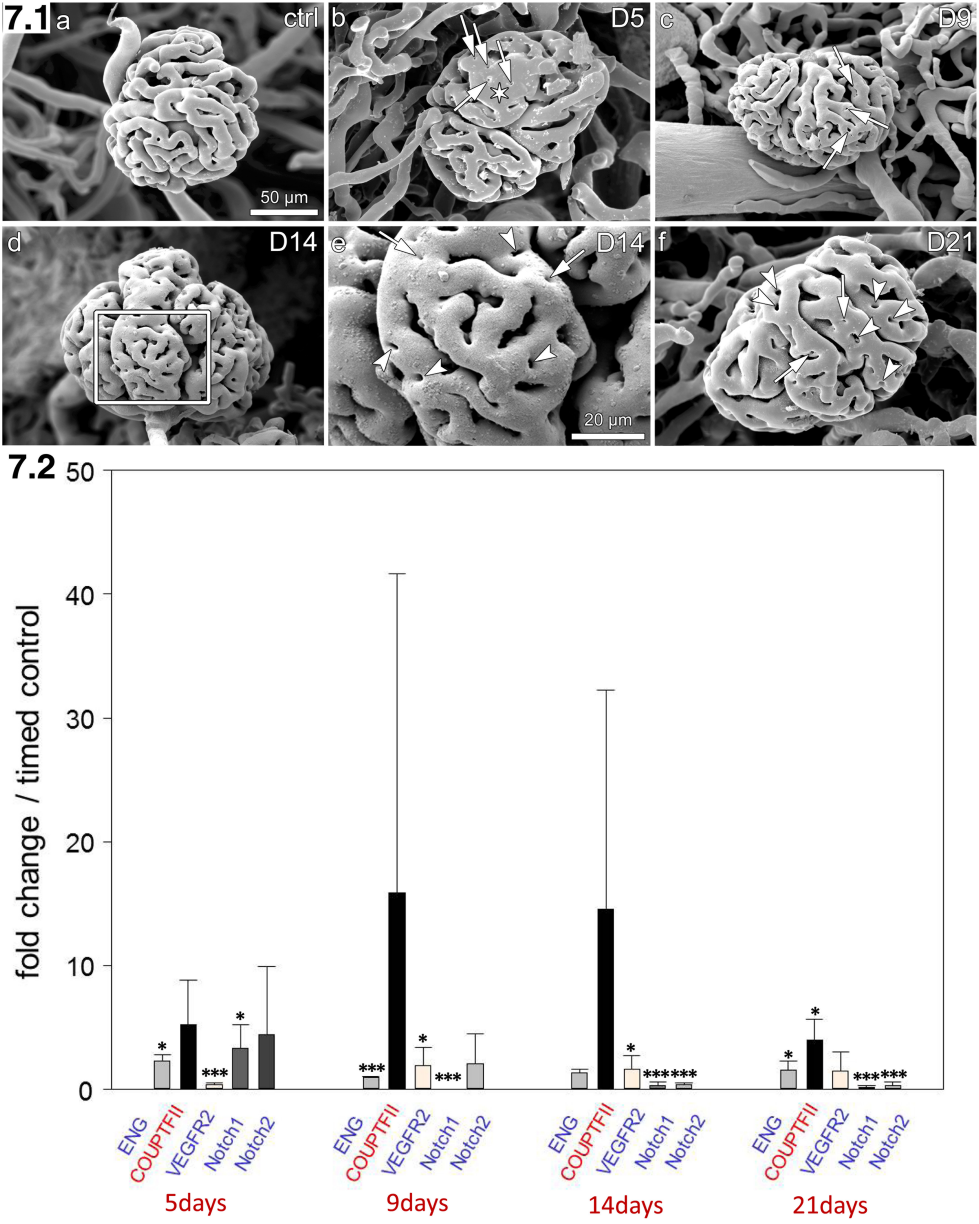
**7.1** a-d and f: rat glomeruli casts. a: control animal, b, c, e, f: different stages of Thy1.1nephropathy, e: detail from picture D (day 14 after treatment with anti-Thy1.1 antibody). Indicated are: small holes with arrows (pillars), small capillary loops with arrowheads, asterisk for glomerular microaneurysm at D5. **7.2**: Fold change in gene expression of ENG, COUP-TFII, VEGFR2 and Notch in the kidney cortex of rats with Thy1.1 nephropathy compared to healthy control group (n = 4). Below the time course in days is indicated.

Next figure (Fig 7.1) presented SEM pictures of rat glomeruli at different stages of nephropathy originated from treatment with anti-Thy1.1 antibody. This can be considered as visualization of time course of gene expression pattern shown in Fig 7.2.

## Discussion

**Fig 8:**
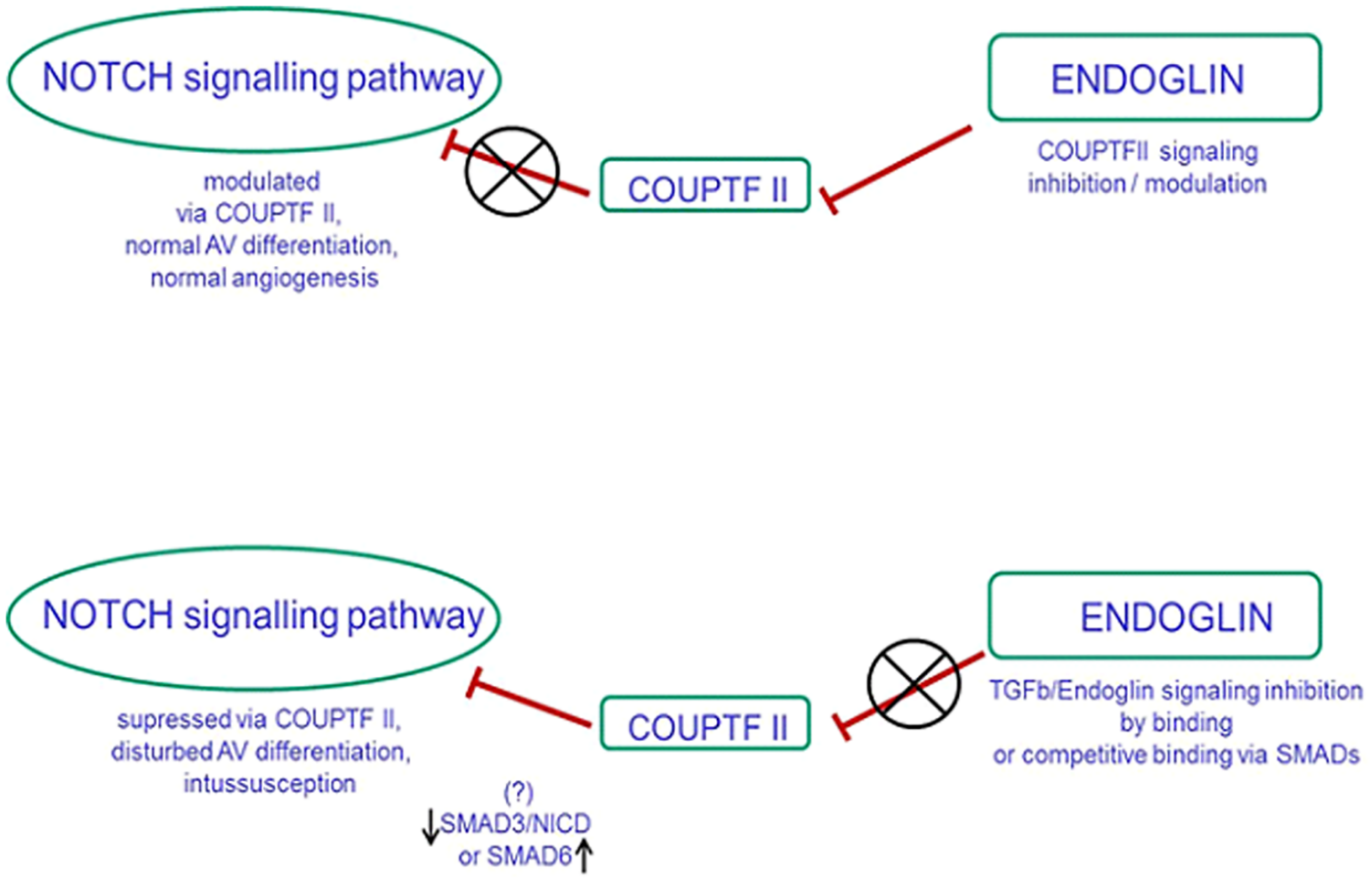
Proposed model for interaction between ENG, NOTCH and COUPTF II.

In the present study, we show that ENG inhibition influences expression of the chicken ovalbumin upstream promoter transcription factor and thus promotes intussusceptive angiogenesis. These data are consistent with previous reports [16] showing that absence of ENG influenced expression of COUP-TFII. Currently it is not completely clear, how ENG affects COUP-TFII, but based on the data obtained so far it is plausible that ENG interaction with TGFβ /SMAD proteins is a link to COUP-TFII acting as transcription modulator (Fig 8).

In agreement with the results reported here, other reports described in different systems, an indirect association between the TGFβ and the Notch pathway. During normal vascular development Notch triggers arterial- and COUP-TFII venous- (in which it inhibits Notch signaling in the vessels) phenotype. Therefore, inhibition of Notch signaling after pathological blockade of Notch (shown in our previous study) results in a similar vascular pattern as 24 h after ENG inhibition, where significant increase in the size of the vascularized area and pillar amount can be detected. Also observed relatively often in this study IA occured on the arterial side, whereas during normal conditions is predominant on the venous side of vasculature. The latter observation supports the idea that these factors are important not only for induction of arterial specification at the early stages of development but are as well requisite for maintenance of mature vasculature and also play a role in IA. Endoglin regulates proliferation of endothelial cells and promotes expansion and maturation of vascular network [15, 16] as well as prevents development of vascular malformation or atypical boundaries between arteries and veins. Conditional deletion of ENG in endothelial cells of the embryo and postnatal retina indicates that this transcription factor is indispensable for a correct acquisition and maintenance of arterial identity [32, 33]. Elsewhere, global deletion of ENG was shown to induce major abnormalities in the embryonic vasculature [34], consistent with the observation reported here by visualization of vessel networks in the chicken area vasculosa. Furthermore, as part of the TGFβ receptor complex, ENG was already investigated in some preclinical and clinical studies dealing with anti-cancer therapies. For instance, everolimus therapy correlating with ENG downregulation in breast, pancreatic and urogenital tract tumors seems to be successful [35]. Ceausu et al. investigated the effects of everolimus on the area vasculosa in regard to early angiogenic and lymphangiogenic events, where endoglin and other proangiogenic factors were downregulated [36]. Moreover, they found that everolimus influences angio- and lymphangiogenesis in a dose-dependent manner. It stimulates pillar development at a low dose and promotes lymphatic endothelial cells differentiation from precursor cells of blood islands at higher concentrations. Their findings are useful for a better understanding of the different and sometimes controversial responses of normal and tumor blood vessels to not only everolimus, but in general to anti-cancer treatment in recent clinical trials.

An important question is ‘what could exactly induce intussusceptive growth?’ The pillar number rose steadily from 6 h after ENG inhibition with the peak at around 24 h post inhibition. COUP-TFII expression was concurrently upregulated. The balance between restoration of ENG appearance and COUP-TFII indicates a likelihood for a link between the two factors at molecular level that is important for pillar formation. Further, gene expression profiles of PDGFb or VEGFR2 were over the time of pillar formation and development constant upregulated. Data originating from gene array related to both ENG and COUP-TFII, as well in the broader context TGFβ/Notch, indicated here rather the role of SMAD components. Pathway restricted SMAD 1, SMAD 5, and SMAD 8 mediate BMP signaling, SMAD 4 is a common partner for the pathway-restricted SMAD. More relevant for our question are TGFβ signals transduced by SMAD 3 and SMAD 6 Since is known that SMAD proteins mediate transcriptional activation or repression depending on their associated partners, SMADs possible interaction with ENG and COUPTF should be taken into account. For instance Blokzijl et al. [37] demonstrated a functional synergism between Notch and TGFβ signaling in the regulation of Hes-1. The findings indicated direct interaction between Notch intracellular domain (NICD) and SMAD 3. Qin et al. [27] demonstrated that COUP transcription factor2 serves as a key regulator to inhibit SMAD4-dependent transcription, and consequently overrides the TGFβ dependent checkpoint for PTEN-null inactive tumors. Overexpression of COUP-TFII in the mouse prostate epithelium cooperates with PTEN deletion to increase malignant progression and produce an aggressive metastasis-prone tumor.

The biologic significance of COUP-TFII in prostate carcinogenesis was documented by patient sample analysis, in which COUP-TFII expression or activity was tightly correlated with tumor recurrence and disease progression, whereas it seemed to be inversely associated with TGFβ signaling. TGFβ stimulation leads to phosphorylation and activation of SMAD2 and SMAD3, which form complexes with SMAD4 that accumulate in the nucleus and regulate transcription of target genes. SMAD 6 interaction with SMAD4 interrupts signaling started from SMAD2 and SMAD3 as competitive partner for SMAD4. In our study, after inhibition of ENG we observed diminished amount of all of SMADs except SMAD6, which leads to the conclusion that this protein could be crucial in IA. To recap, ENG/COUP-TFII-balance under normal conditions regulates angiogenesis and, via modulation of NOTCH signaling pathway, arterio-venous (AV) differentiation. In case COUP-TFII is missexpressed eg. in ENG null embryos, Notch is suppressed and the AV differentiation is disturbed. Based on data obtained in our study we hypothesize competitive SMAD4 binding via SMAD6 and interruption of ENG signaling. Inhibition of ENG may lead to disordered modulation/regulation of COUP-TFII/SMAD6 in way disturbing interaction of SMAD3/NICD and caused development of pillars. In Fig 8 we summarize a proposed model of interaction of these factors.

Although this work does not specifically focus on Thy1.1 glomerulonephritis, but IA, the results obtained in rat model *in vivo* are consistent with the observations reported here from chicken embryos. In Thy1.1 glomerulonephritis from day 5 presence of tiny holes in the vascular casts of glomerular vasculature was observed. Those are the so-called “pillars”, the hallmarks of IA. Between days 9 and 14 number of pillars was increased and until day 21 pillars could still be detected. At the RNA level we found trends of increasing COUP-TFII expression simultaneously with decreased ENG appearance. This underlines the fact that the ENG/COUPTFII balance is an important determinant for intussusceptive growth.

Although our project was primarily focused on IA caused by ENG inhibition, other important questions rose during this study and should be discussed. As shown in Fig 3, the morphological investigations revealed: i) intraluminal accumulation of mononuclear cells which first attached to the vascular wall and in a subsequent step extravasated; as well as ii) pericyte detachment with significantly increased periendothelial space. These events indicate most likely chemoattractant capability and increased vascular permeability. Preliminary data from another study suggest that intussusception is most probably synchronized by chemokine factors for example since the chemotactic factors SDF-1/CXCR4 were upregulated only due to the Notch inhibition [38]. The stromal cell-derived factor SDF-1 binding to its receptor CXCR4 directs migration of progenitor cells into the appropriate sites. Thus mononuclear cells contributed to the formation of transluminal pillars with sustained IA resulting in a dense vascular plexus. SDF-1 is also important in angiogenesis where it promotes vascular endothelial cell migration and induces capillary tube formation. The most exciting finding regarding MNCs in this study, however, is the observation that possibly BMP7 induced the expression of SDF-1, which is upregulated in comparison to another BMPs (BMP2 and BMP 9 are down-regulated, see Fig 4) usually known as regulators of SDF1. This is a very important indication since delivery of SDF-1 may be effective in restoring angiogenesis in individuals with vasculopathies [38].

On the other hand, some studies [39–41] have shown that the aberrant expression of BMPs is linked to prostate cancer progression and bone metastasis. Furthermore it has been shown that CXCR4 protein expression correlates with tumor grade in human prostate cancer and SDF-1 mRNA expression is elevated in metastatic prostate tumors. This emphasizes significance of the balance between different factors in angiogenesis correlated diseases such cancer. In this context, both the TGF-β and SDF-1 pathways are suitable as targets of drug discovery efforts; published data suggest potential benefits in the co-targeting of these pathways.

During early vasculature development many classical molecules are highly expressed and it is not surprising that the effects are not superimposable to those regulated by the physiological concentrations of COUP transcription factor II. Another important variable to consider is the degree of vascular maturation. This suggests that depending on vascular maturation and stability, the response to ENG inhibition/modulation may vary considerably.
